# Advances in application of CRISPR-Cas13a system

**DOI:** 10.3389/fcimb.2024.1291557

**Published:** 2024-02-19

**Authors:** Yue Zhang, Shengjun Li, Rongrong Li, Xu Qiu, Tianyu Fan, Bin Wang, Bei Zhang, Li Zhang

**Affiliations:** ^1^ The Department of Immunology, School of Basic Medicine, Qingdao University, Qingdao, Shandong, China; ^2^ The Department of Clinical Laboratory, Qingdao Women and Children’s Hospital, Qingdao, Shandong, China; ^3^ The Department of Medical Imaging, Shanxi Medical University, Taiyuan, Shanxi, China; ^4^ The Department of Hematology, Taian City Central Hospital, Taian, Shandong, China

**Keywords:** CRISPR-Cas system, CRISPR-Cas13a, genome editing, molecular diagnosis, gene therapy

## Abstract

Clustered Regularly Interspaced Short Palindromic Repeats (CRISPRs) and CRISPR-associated (Cas) proteins serve as an adaptive immune system that safeguards prokaryotes and some of the viruses that infect prokaryotes from foreign nucleic acids (such as viruses and plasmids). The genomes of the majority of archaea and about half of all bacteria contain various CRISPR-Cas systems. CRISPR-Cas systems depend on CRISPR RNAs (crRNAs). They act as a navigation system to specifically cut and destroy foreign nucleic acids by recognizing invading foreign nucleic acids and binding Cas proteins. In this review, we provide a brief overview of the evolution and classification of the CRISPR-Cas system, focusing on the functions and applications of the CRISPR-Cas13a system. We describe the CRISPR-Cas13a system and discuss its RNA-directed ribonuclease function. Meanwhile, we briefly introduce the mechanism of action of the CRISPR-Cas13a system and summarize the applications of the CRISPR-Cas13a system in pathogen detection, eukaryotes, agriculture, biosensors, and human gene therapy. We are right understanding of CRISPR-Cas13a has been broadened, and the CRISPR-Cas13a system will be useful for developing new RNA targeting tools. Therefore, understanding the basic details of the structure, function, and biological characterization of CRISPR-Cas13a effector proteins is critical for optimizing RNA targeting tools.

## 1 Introduction

Gene editing technology also known as genome editing technology refers to the change and correction of DNA sequence and, the destruction of the function of toxic or inhibitory genes (or restoring the function of necessary genes), to achieve genetic therapy (, 2021). Gene editing technology can be divided into three generations, Zinc-Finger Nucleases (ZFN) technology (Miller et al., 2007), Transcription Activator Like Effectors (TALEs) technology (Moscou and Bogdanove, 2009), and CRISPR-Cas technology (Verma et al., 2022). The molecular structure of these three generations of gene editing tools is characterized by non-specific endonuclease fusion of specific DNA recognition domains. In genome modification, artificial nucleases are used to cleave DNA. Three nuclease gene editing tools, ZFN, TALEN, and CRISPR-Cas, are applied in molecular biology to induce double-strand breaks (DSBs) of DNA and stimulate non-homologous DNA repair.

Gene editing technology has been developing rapidly, among which CRISPR-Cas technology is considered to be the easiest and most powerful gene editing tool. Compared to the complex operation and high cost of ZFN and TALEN, CRISPR technology eliminates the tedious process of synthesizing and assembling protein modules that recognize DNA, and its guide RNA (gRNA) design and synthesis work is much less than the previous two and has low cytotoxicity (Zhao et al., 2022). Therefore, it has been widely applied immediately once introduced.

In 1987, a special repeating short palindromic sequence, the CRISPR sequence, was first identified in the genome of *Escherichia coli* (Ishino et al., 1987). In 2000, scientists discovered that this particular sequence is widespread in other bacteria and archaea (Sorek et al., 2008). In 2002, scientists officially named this particular sequence the CRISPRs and Cas genes (Jansen et al., 2002). In 2005, it was found that the spacer sequences in CRISPR were derived from foreign genes, and it was speculated that they might be related to the “adaptive immunity” of prokaryotes. In 2007, the acquired immunity to the CRISPR-Cas system was first demonstrated in experiments.

The CRISPR-Cas system is also known as the adaptive immune system of prokaryotes (Mohanraju et al., 2016). Spacers serve as “immune memory” for prokaryotes in CRISPR-Cas systems, and they play an important role in adaptive immunity since they store the memory of specific Mobile genetic elements(MGEs) encounters acquired by the microbe from previous unsuccessful infections (Datsenko et al., 2012). This immune memory allows the invader to be recognized and neutralized in subsequent infections.

**Figure 1:**
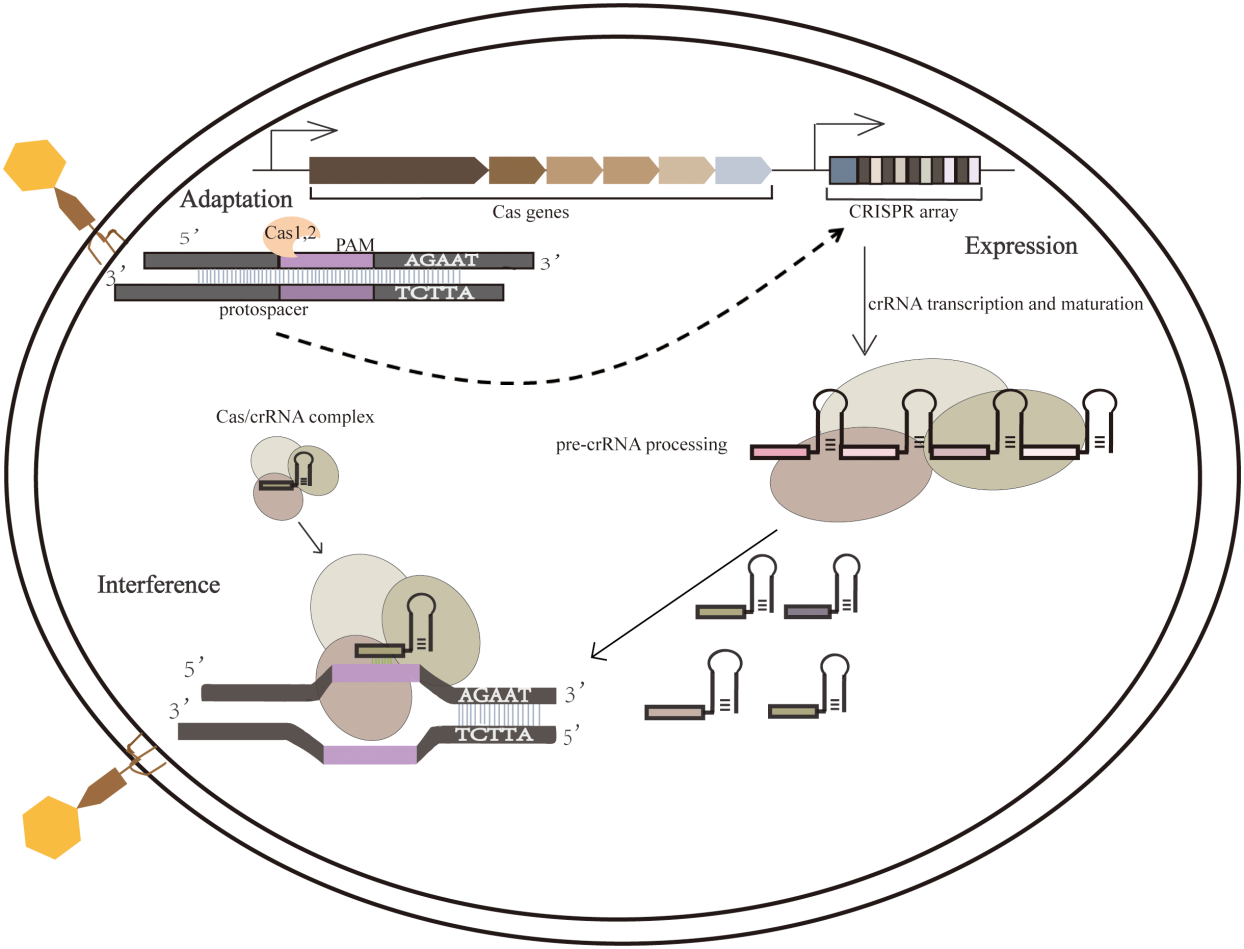
Three phases of the CRISPR-Cas system. During the adaptation phase, when a foreign nucleic acid substance invades, the CRISPR-Cas system integrates it into the CRISPR sequence. During the expression phase, the expression of CRISPR loci containing spacer sequences produces a long primary CRISPR transcript (pre-crRNA). When pre-crRNA becomes mature crRNA, it binds to the Cas protein complex (Cascade). In the interference step, crRNA guides the Cascade complex to a target containing complementary DNA or RNA, cutting the invading DNA or RNA.

Processes of the adaptive immune system consist mainly of the integration of external invading elements into the CRISPR array, which is then transcribed into pre-CRISPR RNAs (pre-crRNAs) and processed into short crRNAs to guide Cas proteins within the crRNA complex targeting other invading homologous DNA. The CRISPR-mediated adaptive immune process consists of three main phases, namely adaptation, expression, and interference. (1)In the adaptation phase, the foreign DNA fragments were processed as new spacers for integration into the CRISPR array; (2)The expression phase refers to transcribing the CRISPR array and then processing the precursor transcripts into mature crRNA, which is then assembled with one or more Cas proteins to form a CRISPR ribonucleoprotein Complex (Zhou et al., 2020);(3)The third phase is interference phase involves directed cleavage of invading homologous viral or plasmid nucleic acids (Takeuchi et al., 2012) (**Figure 1**).

**Figure 2:**
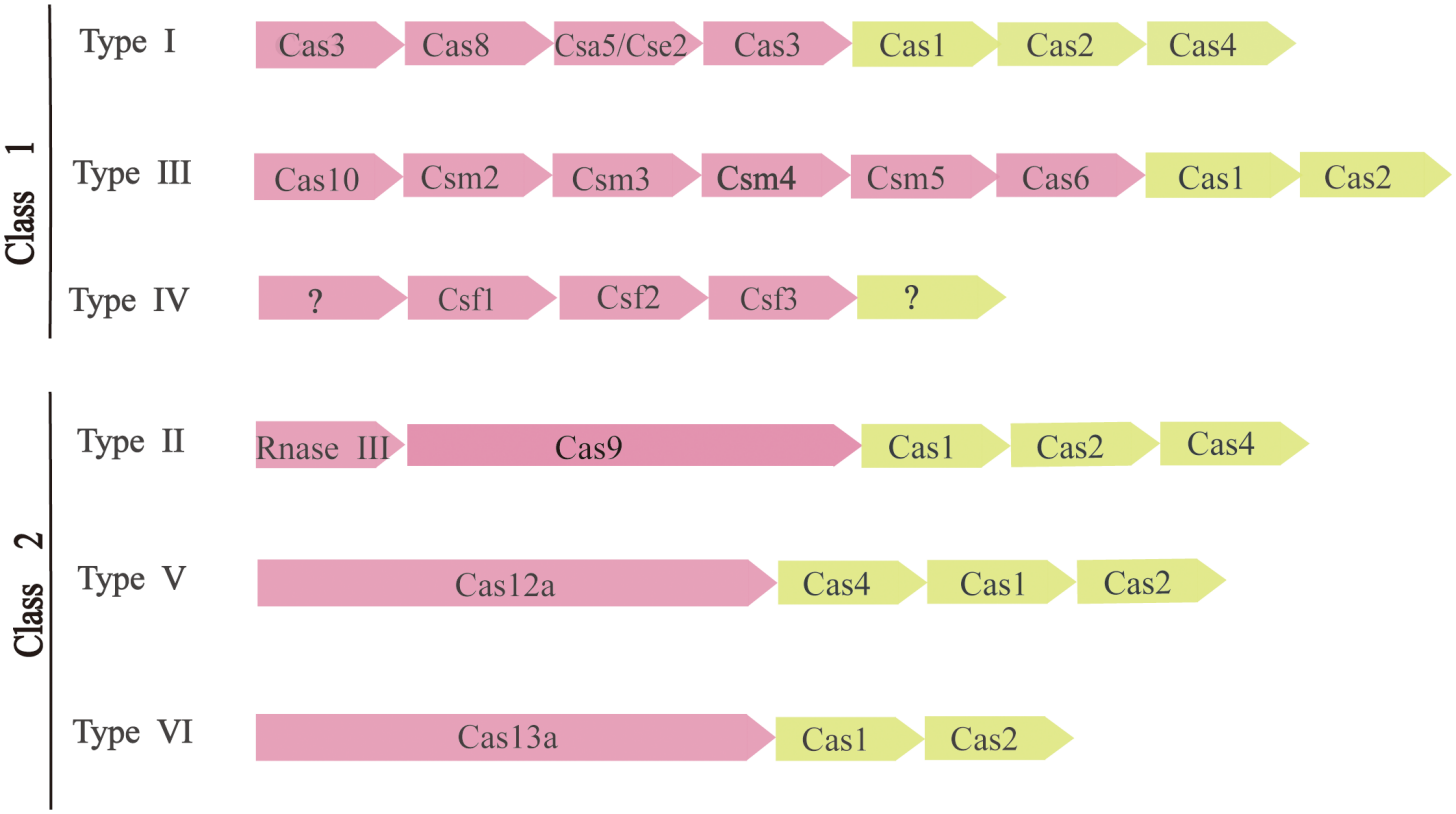
Composition and classification of CRISPR-Cas system. The modules represent CRISPR-related transposons. Pink represents the expression and interference stage, and light yellow represents the conservative adaptation stage. Question marks indicate components that are currently unknown.

To survive, bacteria and archaea have evolved a vast variety of anti-virus defense mechanisms. The defense system also includes receptor masking, restrictive modification (R-M) systems (Ershova et al., 2015), DNA interference (Argonaute) (Kuzmenko et al., 2020), and phage rejection (BREX or PGL) (Goldfarb et al., 2015). A kind of arms race between the CRISPR-Cas system and external invasive material usually refers to the rapid evolution of external invasive genetic material and Cas genes (Takeuchi et al., 2012). As a result of the ongoing exogenous nucleic acid-host arms race, CRISPR-Cas system rapidly evolved through the horizontal transfer of intact sites or individual modules, with structural and functional diversity. There are many ways to classify the CRISPR-Cas system (Makarova et al., 2011). According to the latest CRISPR-Cas systematic classification method and the characteristics of Cas proteins, There are two main classes of CRISPR-Cas systems, each of which is further subdivided into three (**Figure 2**).

The apparent modularity of CRISPR-Cas loci is a key feature of organization and evolution. Different CRISPR-Cas systems have largely consistent and conservative adaptation modules. It consists of Cas1 (Wiedenheft et al., 2009) and Cas2 (Beloglazova et al., 2008) genes, both of which are required to acquire spacers. The adaptation module also includes the Cas4 gene in many CRISPR-Cas variants. In contrast, CRISPR-Cas effector modules involved in crRNA maturation and target recognition and segmentation showed greater versatility (Mohanraju et al., 2016).

The two types of CRISPR-Cas systems are fundamentally different in the effects module. The Class 1 CRISPR-Cas system is considered to be the most primitive ancestral system before evolution. Class 2 systems evolved from Class 1 systems by inserting transpositive elements that encode all kinds of nucleases, it is now one of the tools used for genome editing. The main difference between them is the composition of Cas proteins. Class 1 CRISPR-Cas systems, which include types I, III, and IV, are mainly found in bacteria and archaea and contain effecting complexes composed of four to seven multiple Cas protein subunits, mainly multi-protein effector complexes. Class 2 CRISPR-Cas systems, including types II, V, and VI, occur almost entirely in bacteria, and effector complexes consist of single-protein effector complexes (Mohanraju et al., 2016).

CRISPR-Cas13a system targeted with a programmable RNA based on a single component contains two higher eukaryotic and prokaryotic nucleotide-binding (HEPN) domains, often associated with Ribonucleases (RNases), indicating RNA-directed RNA targeting function (Abudayyeh et al., 2016). Regulation of gene expression or alteration of gene transcripts at the RNA level in living cells provides a key regulatory manner. Editing or partially destroying transcripts is a unique approach to analyzing gene function, providing both complementary benefits to classical genetic methods and new insights into the regulation of gene function at the RNA level.

New CRISPR-Cas systems revealed by bioinformatics approaches further expand the CRISPR “universe” to include types V and VI, as well as several subtypes. Several recent studies have not only classified new CRISPR-Cas systems but also highlighted that CRISPR-Cas-driven immune mechanisms are more complex and subtle than previously recognized. For example, recent studies have highlighted the role of Cas10 “second messenger” generation amplification of antiviral responses in controlling Csm6 activity and effector proteins such as Csm6 and Csx1 (Smargon et al., 2017). Together, these studies suggest that prokaryotes have evolved ways of tweaking their antiviral responses, possibly to maintain horizontal gene transfer that allows for potentially beneficial traits.

## 2 Overview of CRISPR-Cas13a

CRISPR-Cas13a is an RNA-directed RNA-targeting nuclease belonging to Class 2 type VI CRISPR-Cas system (Abudayyeh et al., 2016). The CRISPR-Cas13a system is widely used because it targets RNA rather than DNA and puts less pressure on cell survival. Cas13a is unique in the ribonuclease and can completely degrade single-stranded RNA. Activated Cas13a can not only cut RNA-containing sequences complementary to the guide but also cut RNA-free in solution. Cas13a also contains catalytic sites for RNA-directed target cleavage, but these sites are located on the outer surface. Thus, once activated by binding to target RNA, it can be used as an ordinary non-specific RNase to cut any available mRNA.

CRISPR-Cas13a recognizes target RNA through the 3’ proto-spacer flanking sites (PFS) (Tang and Fu, 2018). Cas13a recognizes single-stranded RNA target sequences and activates RNase activity catalyzed by the ribonuclease domain. Its target RNA recognition relies on the presence of one non-G nucleotide in 3’ PFS (Liu et al., 2017a). Upon activation, Cas13a degrades not only target RNA but also “collateral” RNA, suggesting that, unlike other target nucleic acids that are cis-cleaved and bound by Class 2 CRISPR-related nucleases, Cas13a trans degrades its RNA substrate (Bot et al., 2022). The specificity of the CRISPR-Cas13a system can be improved by designing hairpin spacer crRNAs (hs-crRNAs) to inhibit the binding affinity between Cas13a and off-target RNAs (termed the hs-CRISPR system) (Ke et al., 2021).

When the phage attacks the host, the crRNA’s guide region forms a double-stranded structure, directing Cas13a to switch to a catalytically active state and make a disordered cut of any exposed single-stranded RNA. This suicidal attack on its RNA is combined with a direct attack on viral RNA. This finding is consistent with the results that the expression of Cas13a protein, crRNA, and target RNA causes cytotoxicity (Abudayyeh et al., 2016).

### 2.1 Composition of CRISPR-Cas13a

**Figure 3:**
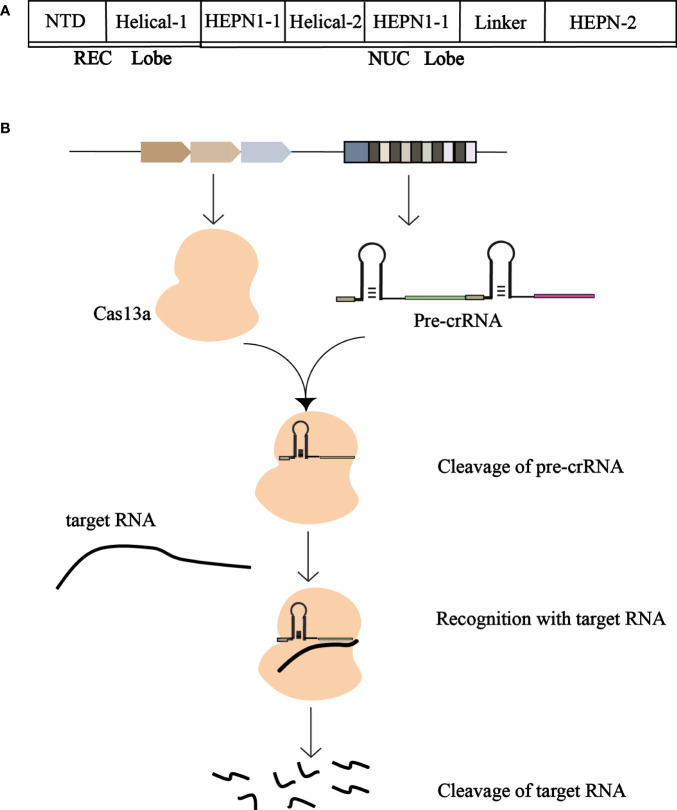
**(A)** Schematic representation of the structure of Cas13a. The REC and NUC lobes form Cas13a. The REC lobe consists of a Helical-1 domain and an N-terminal domain, and the NUC lobe consists of HEPN1, HEPN2, Helical-2, and a Linker. **(B)** Schematic of the working mechanism of CRISPR-Cas13a. Cas13a cleans pre-crRNA to produce mature crRNA, which binds to mature crRNA to generate CRISPR-crRNA monitoring complex to recognize the target RNA and degrade the foreign target RNA.

Cas13a has two conserved HEPN domains (nucleotide-binding domains of higher eukaryotes and prokaryotes). These two domains are typically involved in the cutting of targeted mRNAs with complementary sequences for crRNA-guided recognition (Mahas et al., 2018). The structure of the Cas13a is a two-leaf structure consisting of a recognition (REC) lobe and a nuclease (NUC) lobe (**Figure 3A**). The REC flap consists of the N-terminal domain (NTD) and Helical-1 domains, and the NUC flap includes two conserved HEPN domains and a Linker and Helical-2 domains (Liu et al., 2017c). The Dual RNase activity model of Cas13a has two interdependent RNase activities with two independent catalytic sites. The RNA cutting site on the outer surface of Cas13a is composed of two HEPN domains. The two RNase catalytic sites are physically distant from one another, enabling it to cleave RNAs promiscuously and process pre-CRISPR RNAs specifically. The Helical-1 and HEPN2 domains are responsible for the stabilization and processing of pre-crRNA.

Cas13a cleaved the precursor crRNA to produce mature crRNA, which was then combined to form the Cas13a-crRNA monitoring complex. The crRNA repeats contain raised stem rings fixed to the REC leaves. There is a gap between the Helical-1 and HEPN2 domains in the 5’ side region of the crRNA repeat, crRNA preconditioning be mediated by Helical-1 domains and HEPN2 domains (Liu et al., 2017b).

### 2.2 Mechanism of CRISPR-Cas13a

When foreign nucleic acids invade the host, Cas13a cutting pre-crRNA generates mature crRNA and binds with mature crRNA to produce a CRISPR-crRNA monitoring complex. The crRNA’s guide region recognizes and complements the target RNA, allowing Cas13a to degrade the RNA (**Figure 3B**). The formation of a guide target duplex activates Cas13a. When bound to the target RNA, further conformational changes occur in Cas13a and crRNA. The binding of target RNA to crRNA induces a conformational change in Cas13a, forming a crRNA-target RNA double-binding channel (Wiedenheft et al., 2009). During the formation of the Cas13a- crRNA-Target ternary complex, the Helical-2 and HEPN1 domains undergo obvious conformational changes, while the NTD, Helical-1, and HEPN2 domains are well aligned. The Helical-2 domain rotates away from the HEPN2 domain, while the HEPN1 domain rotates toward the HEPN2 domain. In addition, moderate migration occurred between the ligand subdomain and the HEPN2 domain. These rearrangements of the Helical-2, HEPN1, and Linker domains generate a wide binding channel for the crRNA-target RNA duplex domain relative to the HEPN2 domain. The crRNA and the target RNA form a 28 bp guide-target RNA double strand, which is very close to the A-type Helical structure, located in the central channel within the NUC leaf. The interaction between the target RNA and Cas13a is essential for crRNA-directed RNA cleavage. Stem loops of crRNA repeats are anchored in cracks formed between the NTD and Helical-1 domains, forming extensive contacts between crRNA and proteins (Bot et al., 2022).

At the same time, conformational changes were also observed in crRNA, especially in the crRNA repeat sequence and the 3’ region, and new interactions were formed with Cas13a. the crRNA binding Cas13a had a stem ring containing 2nt protrusion, indicating that the stem ring with 2nt protrusion played an important role (Liu et al., 2017b). The length of the spacer and the number and location of the spacer mismatch with the target RNA are critical for activating the RNase activity of Cas13a. To maintain the cracking capacity of Cas13a, the interval length is required to be at least 20nt (Abudayyeh et al., 2016). In effect, the target RNA is an activator that activates two catalytic sites within the HEPN domain by forming a double with the crRNA’s guide region and bringing the catalytic residues close together. Activated HEPN catalyzes the cleavage of ssRNA at the site. Cas13a has two independent catalytic sites for its two interdependent RNase activities.

The CRISPR-Cas13a system has been widely used to detect and treat human diseases. It can be used for bacterial detection, antibacterial agents and bacterial gene detection, viruses, such as antiviral infections, and eukaryotic organisms, such as yeast and plants.

## 3 CRISPR-Cas13a application

CRISPR-Cas13a is an RNA-guided RNA ribonuclease and has a wide range of applications in RNA editing technology.

**Figure 4:**
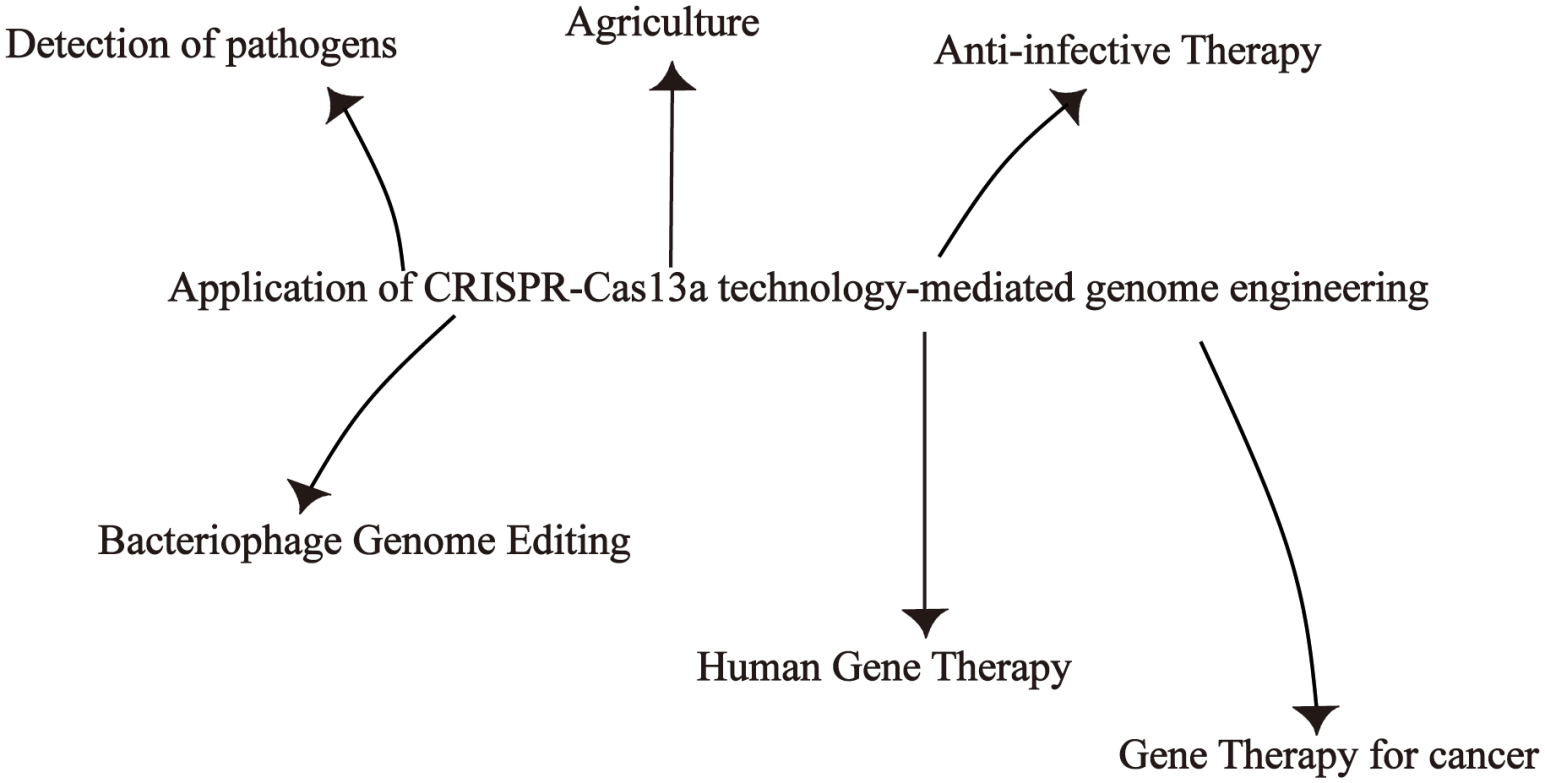
Application of the CRISPR-Cas13a system.

The discovery that Cas13a effector is characterized by collateral RNA cleavage activity has led to greater interest in developing novel biosensing technologies for nucleic acid detection and holds the promise of significant advances in CRISPR diagnostics (Zhao et al., 2022). CRISPR-Cas13a systems are used to detect a variety of pathogens, such as bacteria, viruses, and cancer cell detection. In addition to gene editing functions, the CRISPR-Cas13a system can also be used for phage genome editing. The use of CRISPR-Cas13a in plants and fungi also has the potential to accelerate the pace and process of agricultural research. More and more studies have shown that CRISPR-Cas13a can also be used in tumor therapy and gene therapy (**Figure 4**).

### 3.1 Detection of pathogens

CRISPR-Cas13a biosensors have become a hot topic in the field of molecular diagnostics, and have been used to detect various targets^29^. Many studies have shown that the CRISPR-Cas13a system, as a new tool for molecular diagnosis, is expected to improve the breadth, sensitivity, and specificity of molecular diagnostic tools such as antigen and antibody reaction, PCR detection, and genome sequencing.

#### 3.1.1 Bacterial detection

Food contamination and some serious diseases caused by bacteria and their toxins place a huge burden on public health. *Staphylococcus* is a major member of the human skin microbiome and one of the most common causes of hospital infections. The rate at which methicillin-resistant and vancomycin-resistant *Staphylococcus aureus* (MRSA and VRSA, respectively) are increasing in hospitals and the community has far exceeded the level of new antibiotic discovery, and the main reason for this problem is the transfer of antibiotic resistance and virulence genes through binding to plasmids and other mobile genetic elements (Zhou et al., 2020).

As a sensitive, rapid, and specific technology, the CRISPR-Cas13a system is increasingly used in bacterial detection. A bacterial sensing strategy named CCB-Detection (CRISPR-Cas13a-based bacteria Detection) has been developed using the CRISPR-Cas13a system. That is the “side effects” of crRNA programmability and Cas13a hybrid RNase activity on target RNA recognition. By selecting *Staphylococcus aureus* as the model bacterium, CCB-Detection has been verified that it can be used to detect *Staphylococcus aureus* and meet our needs. The detection range of CCB-detection was 10^0^-10^7^ CFU/mL, and the sample reporting time of CCB-detection was less than 4 hours (Zhou et al., 2020). The selectivity of CCB for *Staphylococcus aureus* was satisfactory and there was no interference from other bacteria. This method has been successfully applied to the detection of Staphylococcus aureus in real food samples, and its performance is superior to the gold standard culture counting method.

The CRISPR-Cas13a system can be used to detect the virulence of *Yersinia pestis* (Schultzhaus et al., 2021). The test uses the CRISPR-Cas13a system for sensitive and specific recognition of the sequence. This includes detailing the diversity of crRNA properties, identifying assay sequences with atomic molar sensitivity and specie-level specificity, and proposing a method to simply simplify the crRNA screening process to allow future development of the high-throughput tests required for analytical design rules (Haurwitz et al., 2010).

Several studies have achieved the detection of mutants of resistant strains of different pathogens (Wessels et al., 2020). For example, the researchers built a phage capsid containing a programmed Cas13a system that targets genes from drug-resistant strains of *E. coli.* Like the susceptibility test, *E. coli* treated with CRISPR-Cas13a that carried genes from the resistant strain did not grow in Petri dishes. Experiments have shown that target resistance genes located on the plasmid or chromosome can be accurately detected. The construction of CapsidCas13a further confirms the detection capability of the method, showing that the method is suitable for detecting any bacterial gene regardless of its location on the bacterial chromosome or plasmid (Kiga et al., 2020).

While CapsidCas13a can detect genes on chromosomes and genes on plasmids, optimized CapsidCas13a(s) can also effectively detect toxin-coding genes and distinguish different genes located on the same plasmid, which is also suitable for clinical isolates.

#### 3.1.2 Virus detection

The COVID-19 pandemic, caused by the transmission of SARS-CoV2, requires fast, sensitive, and inexpensive tests to aid diagnosis. Nucleic acid testing facilitates pathogen detection and disease course monitoring (Hussein et al., 2022). Therefore nucleic acid test as the gold standard has been widely promoted. However, due to the limitations of machines and professionals, the simplicity, cost, and specificity of nucleic acid testing cannot meet the requirements, which leads to a series of problems such as prolonged results and incidental problems (such as isolation time, and transmission range) (Wessels et al., 2020). RT-qPCR, the current gold standard test used for SARS-CoV-2 gene testing, has a long turnaround time, relies on materials and equipment that can be expensive, and is subject to supply chain pressures.

CRISPR-Cas adaptive immune systems contain programmable endonuclease for CRISPR-based diagnostics (CRISPRDx). The CRISPR-Cas13a system is reprogrammed via crRNA to provide a platform for specific RNA sensing (Myhrvold et al., 2018). After identifying its RNA target, activated Cas13a participates in the “indirect” cutting of nearby non-target RNAs. This crRNA-programmed collateral cleavage activity allows Cas13a to detect the presence of specific RNAs *in vivo* by triggering programmed cell death or *in vitro* by marking the non-specific degradation of RNAs. SHERLOCK (Specific High Sensitivity Enzyme Report Unlock) is an *in vitro* report of RNA side cleavage based on nucleic acid amplification and Cas13a-mediated RNA, which achieves real-time detection of targets (Myhrvold et al., 2018).

#### 3.1.3 Cancer cell detection

In tumor diagnosis, compared with traditional tumor detection methods, some early cancer diagnosis methods are more favored by clinicians. For example, free DNA and microRNA in peripheral blood are more conducive to the diagnosis of early cancer.

Cell-free DNA (cfDNA) is the free DNA released by white blood cells or other cells in disease or stress. It is a kind of DNA that is free from cells in peripheral blood (Lanman et al., 2015). Physiologically, cfDNA in blood is mainly derived from necrosis or apoptosis of leukocytes. Studies have shown that the concentration of cfDNA is increased in many solid tumors such as esophageal cancer (Qiu et al., 2018) and lung cancer (Fan et al., 2019), and it has higher sensitivity and specificity compared with traditional tumor markers. The traditional detection methods of cfDNA include gene sequencing technology, including Sanger sequencing, next-generation sequencing, and digital PCR. The second-generation SHERLOCK has been successfully used to detect EGFR L858R mutation in cfDNA samples of non-small cell lung cancer patients.

Extracellular Vesicles (EVs) originate from the intracellular transport system and play an important role in signaling and organelle function interaction (Gruenberg, 2001). EVs contain microRNAs (miRNAs), groups of small non-coding RNAs that regulate protein output after transcription and are predicted to regulate hundreds of targets, thus controlling most protein-coding genes. It also participates in post-transcriptional gene regulation by inhibiting the translation and cutting of target RNA transcripts (Vidigal and Ventura, 2015). In tumor cells, miRNAs are involved in the regulation of tumor cell proliferation. It is known that the complementary pairing of miRNA and target mRNA can regulate the target and then affect the gene expression, and regulate the proliferation, differentiation, and apoptosis of normal cells. Abnormal expression of miRNAs is related to diseases, and miRNAs can be used as potential biomarkers for early diagnosis of tumors. The traditional methods of miRNA level include DNA microarray technology, RNA sequencing method, and RT-qPCR.

A new method for the quantitative detection of EV RNA based on liposome fusion and CRISPR-Cas13a technology has been developed. The CRISPR-Cas13a system was successfully used to detect miR-21-5p in EVs from five different ovarian cancer cell lines (OVCA429, SkOV3, ES-2, CaOV3, and OV90) and one benign cell line (TIOSE4) (Hong et al., 2023). This method simplifies the traditional detection methods without the need for amplification and extraction and improves sensitivity and specificity. Using the CRISPR-Cas13a detection system, miRNA detection can also be achieved. Shan (Shan et al., 2019) et al. used CRISPR-Cas13a to directly detect miRNAs in tumors.

CRISPR-Cas13a system with high sensitivity and high specificity has been applied to the rapid detection of tumor-related markers and targeted therapy of oncogenic mutations and this system has considerable application potential.

### 3.2 Anti-infective function

#### 3.2.1 Anti-bacterial agent

Microorganisms play a key role in human health and environmental nutrient cycles and are often used in a variety of industrial processes. In each case, some strategies can remove some microbes but not others, such as conventional antibiotics and some strain-specific antimicrobial peptides, expression of antibiotic resistance genes, etc. Antimicrobial resistance (AMR) of pathogens has become a growing global public health problem over the past decade (Razavi et al., 2020). In the face of multiple drug resistance and many nosocomial infections, we urgently need a new antimicrobial agent to deal with the current serious situation. With the widespread use of broad-spectrum antibiotics, people have come to realize the importance of targeted killing of certain drug-resistant bacteria to maintain the stability of normal flora. Therefore, identifying resistant pathogens and rationalizing drug use is critical to curbing antibiotic resistance (Yosef et al., 2015).

Horizontal gene transfer (HGT) refers to the transfer of virulence and antimicrobial resistance genes between bacteria, which are the main cause of the emergence of pathogenic strains. CRISPR has been shown to prevent phage infection and plasmid conjugation and constitutes a barrier against all mechanisms of HGT (Bikard et al., 2012). The CRISPR technology makes it possible to design CRISPR antimicrobials with specific bactericidal effects.

In 2014, Bikard et al. and Citorik (Bikard et al., 2014) Et al. developed programmable sequence-specific antimicrobials using phages as “transport carriers” and the delivered RNA-guided nuclease Cas9. The use of programmable Cas9 nucleases as sequence-specific antimicrobials is to manipulate heterogeneous bacterial populations. The CRISPR-Cas9 antimicrobial can be used with antibiotic-resistant bacteria, such as beta-lactam or vancomycin-resistant staphylococcus (Gomaa et al., 2014). Using this new strategy, the researchers were able to selectively target certain strains while maintaining the integrity of others. CRISPR antimicrobials can also be used to remove antibiotic-resistant plasmids from bacteria. Antibiotic-resistant plasmids are plasmids carried by bacteria that protect them from the killing effects of antibiotics (Kim et al., 2016).

The researchers used the CRISPR-Cas13a system to enable crRNA to recognize the promiscuous-RNA cutting ability of the target RNA, resulting in host cell death, resulting in a new class of sequence-specific bacterial antimicrobial agents. In terms of killing efficiency, CRISPR-Cas13a is expected to be better than CRISPR-Cas9 (Kiga et al., 2020). In contrast to CRISPR-Cas9-based antimicrobials, which lack bactericidal ability when the target gene is located on the plasmid, CRISPR-Cas13a shows strong bactericidal activity when recognizing the target gene. Regardless of its location, the Cas13a structure is created by packaging programmed CRISPR-Cas13a into a phage capsid to target antimicrobial resistance genes (Gootenberg et al., 2017).

The study reported the development of an antimicrobial nucleocapsid based on CRISPR-Cas13a, called CapsidCas13a(s). It can specifically kill carbapenem-resistant *E.coli* and methicillin-resistant Staphylococcus aureus by identifying the corresponding sequence of antimicrobial resistance genes.

#### 3.2.2 Antiviral infection

Viruses exist widely in nature and constantly infect bacteria and archaea. Bacteria and archaea have evolved a wide range of antiviral defense mechanisms in response to this pressure. However, because viruses have a high rate of mutation and recombination, they are likely to quickly escape the defenses that bacteria and archaea evolved. As a result, host defenses must also adapt and evolve quickly, leading to a persistent virus-host arms race. The CRISPR-Cas13a system provides us with a new antiviral pathway by targeting viral ssRNA. CRISPR-Cas13a may still be an effective antiviral drug that can kill infected cells.

The development of new antiviral drugs is necessary as viruses mutate to increase their ability to escape immune, especially in the face of outbreaks of Zika virus, Ebola virus, the current SARS-CoV-2 pandemic, and a possible influenza pandemic in the future. However, current antiviral drugs are mainly embodied in small-molecule antibodies and neutralizing antibodies, which are concentrated in the need for high doses or frequent read ministration to obtain functional effects. It is therefore critical to address the need for antiviral drugs that are broad- spectrum, flexible, and effective across multiple virus species or strains.

CRISPR-Cas13a system was released using a nebulizer currently used in humans to simulate therapeutic strategies and demonstrate that CRISPR-Cas13a system can effectively degrade influenza viral RNA *in vivo*. Subsequently, the effectiveness of CRISPR-Cas13a system against SARS-CoV-2 infection was evaluated in a Syrian hamster model and demonstrated that a single dose was sufficient to alter the pathophysiology of infection (Blanchard et al., 2021). This work demonstrates great progress in the use of Cas13a to treat respiratory viral infections. Direct detection of SARS-CoV-2 RNA with CRISPR-Cas13a system and mobile phones provides a promising option for rapid, point-of-care detection of pathogens (Fozouni et al., 2021). In the future, CRISPR-Cas13a system will be used in more antiviral treatments and detection.

Gootenberg et al. developed SHERLOCK version 2 (SHERLOCKv2) by improving on the original approach. The method combines CRISPR-Cas13a-based detection with four multiplexed channels to achieve instrument-free detection of DENV or ZIKV ssRNA within 90 min with a sensitivity of 2 aM (Zhang and You, 2020). The symptoms of the disease caused by Ebola are ambiguous, posing a great challenge for diagnosis. Hence sensitivity and immediate detection methods are needed for Ebola. A new method called Cas-Roller, which combines the Cas13a reaction with the DNA roller reaction to detect Ebola RNA, has been reported and achieved 291 aM LOD in 40 minutes.

The latest research demonstrates that CRISPR-Cas13a, as an adaptive immune system, not only reduces the levels of newly synthesized viral RNA but also targets and destroys viral RNA that enters the cell within the viral capsid, leading to strong inhibition of HIV-1 infection.

### 3.3 Phage genome editing

Recent research suggests that the potent broad-spectrum antiviral activity of Cas13a can be used as a sequence-specific deselection system suitable for restoring minimally edited phage variants with a single codon replacement (Adler et al., 2022). CRISPR-Cas13a system was applied to editing broad-spectrum phages. Two-step methods, homologous recombination and enrichment methods were used. Editing in the genome of phages introduced by homologous recombination can evade CRISPR-Cas13a system targeting, while wild-type phages cannot. The study achieved seven marker-free genome edits in three different phages with 100% efficiency.

The research shows that CRISPR-Cas13a can be used as a universal tool for editing the richest and most diverse biological entities on Earth. This study highlights the vulnerability of phage RNA molecules during phage infection and provides a powerful general strategy for phage genome engineering (Varble et al., 2021).

### 3.4 Agriculture

#### 3.4.1 Botany

RNA is involved in almost all biological activities and plays an important role in the biological world. The development of tools to manipulate the transcriptome is therefore crucial. The widespread importance of RNA has led to the development of innovative approaches to RNA transcription processes, such as RNA interference, to regulate RNA transcriptional abundance to control viral infection. Improve plant survival in adverse environments by altering epigenetic states and increasing transcriptome plasticity. CRISPR-Cas13a can target specific endogenous and viral RNAs in mammalian cells and plants (Ali et al., 2018). Plant RNA viruses are a major cause of commercially important plant diseases, infecting a wide range of plant species and causing severe quality and quantity losses in a wide range of key crops. Recent results suggest that the CRISPR-Cas13a system is also suitable for fighting plant viral infections (Wolter and Puchta, 2018). Because single-stranded RNA viruses are by far the most abundant virus class in plants, the CRISPR-Cas13a system could do the most good. The most complex changes in plant metabolism can be achieved by combining DNA and RNA targeting systems.

Pottyviruses are plant-infected viruses belonging to the genus Pottyviruses, which is one of the largest groups of plant viruses besides begomovirus. Potato virus is a kind of pathogen of important economic significance, which can infect many crops and cause serious damage to them. The study revealed that the catalytic activity of CRISPR-Cas13a interferes with Tumv-GFP transient experiments and Bunson tobacco stable overexpression lines. Cas13a can process long pre-crRNA transcripts into functional crRNA, which leads to TuMV interference (Aman et al., 2018). By studying the expression of FnCas9 and LshCas13a, an efficient transgenic construct screening system was established to inhibit grape leaf roll-associated virus 3 (glav-3), one of the pathogens causing grape leaf roll disease (GLD) (Jiao et al., 2022).

The results suggest that Cas13a is an RNA-directed ribonuclease that can be programmed to target and degrade viral RNA genomes, thus providing a promising and effective tool for a variety of RNA manipulations, particularly interference with RNA viruses in plants and eukaryotic cells.

#### 3.4.2 Yeast

Alteration of gene transcripts at the RNA level in living cells is a key regulatory mechanism. Thus, editing or partially destroying transcripts can be used for gene analysis studies, providing complementary benefits to classical genetic methods and potentially providing novel approaches to gene function regulation at the RNA level (Abudayyeh et al., 2016). The realization and reuse of transcripts were achieved by manipulating genes in fission yeast using the Type VI CRISPR system. Studies have shown that manipulating transcripts has many advantages over manipulating genes: altering gene transcripts does not change the genes themselves, making the changes reversible and controllable in both time and space. This approach is more efficient at targeting genes with multiple copies, especially in polyploidy organisms.

The CRISPR-Cas13a system was implemented in yeast and Cas13a was overexpressed using a plasmid to target and knock down endogenous gene transcripts in fissile yeast cells. A new programmable toolset has been introduced in fission yeast for transcriptome manipulation and is widely applicable to basic genetic and biotechnology research (Lewis and Ke, 2017). The knockdown effect of CRISPR-Cas13a system was investigated by introducing crRNA targeting endogenous gene transcripts. Studies have shown that the presence of crRNA targeting the tdh1 transcript (crRNA-TDH1) results in significantly slower growth of yeast cells than yeast carrying Cas13a alone (Jing et al., 2018). The implementation of the CRISPR-Cas13a system in fission yeast is a stepping stone towards a promising new family of targeted gene transcription tools for a wide range of research and biotechnology applications. It was also demonstrated that the binding of Cas13a-crRNA to target RNA was highly specific and selective, and dCas13a (R1278A mutation Cas13a) showed a stronger binding affinity for its target RNA than the wild-type Cas13a (Jing et al., 2018). This high binding affinity makes Cas13a suitable for a range of applications, such as labeling and RNA modification for molecular localization and subcellular transport.

### 3.5 Human gene therapy

#### 3.5.1 Muscular dystrophy

Amplification of unstable short repeats in the human genome leads to a range of neurological and neuromuscular diseases. Although these diseases differ in symptoms and pathology, they do have overlapping molecular mechanisms, namely the accumulation of amplified RNA. Dilated RNA can induce cytotoxicity by isolating various RNA-binding proteins for RNA function or by translating into dilated proteins for protein function. Amplification of unstable short repeats in the human genome leads to a range of neurological and neuromuscular diseases, such as myotonic dystrophy types 1 and 2 (DM1/2). DM1 is a single-gene disorder, the most common adult-onset muscular dystrophy, for which there is currently no effective treatment (Thornton, 2014).

The use of genome editing to treat single-gene diseases has become increasingly popular in recent years. DM1 is the most common form of adult-onset muscular dystrophy for which there is no treatment. DM1 is a genetic disorder linked to the DMPK gene that causes muscle loss, difficulty breathing, and early death. Because DNA editing with CRISPR-Cas9 is hampered by large deletions and rearrangements, the use of an alternative strategy based on reprogrammed RNA may provide important therapeutic value in treating DM1 diseases. Cas13a belongs to the Type VI CRISPR-Cas system and is an RNA-guided RNase with multiplexing and therapeutic potential. By utilizing the human codon-optimized LshCas13a protein, the aggregation of toxic RNA was tracked and degraded with repetition-based crRNA. Repeated CUG cleavage was observed in DM1 patient-derived myoblast lines by lentivirus delivery (East-Seletsky et al., 2016). Decreased CUG RNA foci and decreased DMPK RNA were observed in the transduced DM1 myoblast line (Zhang et al., 2020).

Studies have shown that Cas13a holds great promise for regulating repeat RNA levels. As our understanding of the selection of crRNA and different Cas13 proteins grows, the CRISPR-Cas13a strategy may be extended to treat other microsatellite expansion diseases.

#### 3.5.2 HIV

Caused by infection with HIV, a virus that attacks the body’s immune system and causes acquired immune deficiency syndrome (AIDS). HIV mainly attacks the most important CD4T^+^ lymphocytes in the human immune system, thereby destroying a large number of cells and causing the body to lose immune function. Therefore, it causes low immune function, prone to various diseases and malignant tumors, and high mortality. At present, there is still a worldwide shortage of effective drugs to cure HIV infection.

The study reported the use of the CRISPR-Cas13a system to inhibit human immunodeficiency virus type 1 (HIV-1) infection by targeting HIV-1 RNA and reducing viral gene expression. A strong inhibitory effect of the CRISPR-Cas13a system on HIV-1 infection in human cells was observed experimentally. CRISPR-Cas13a was found not only to reduce the levels of newly synthesized viral RNA, either from transfected plasmid DNA or from viral DNA integrated into cell DNA, but it also targeted and destroyed viral RNA entering cells within the viral capsid, resulting in a strong inhibitory effect on HIV-1 infection (Yin et al., 2020).

These findings suggest that CRISPR-Cas13a offers a potential new tool for the treatment of viral diseases in humans.

### 3.6 Gene therapy for cancer

In addition to detecting tumor markers, the CRISPR-Cas13a system has also been used in cancer therapy. An Oncogene is a kind of gene that is related to the transformation of tumor cells, and its abnormal expression often leads to the occurrence of tumors. CRISPR-Cas13a system can inhibit tumor growth mainly by specifically recognizing and reducing the mRNA expression of oncogenes in cancer cells, resulting in controlled incidental cleavage and inducing programmed cell death of cancer cells. CRISPR-Cas13a is a powerful RNA knockdown tool whose potential use in cancer cells needs further investigation.

#### 3.6.1 Glioma

Glioblastoma multiform (GBM) is the most common malignant brain cancer (Ohgaki and Kleihues, 2005). The prognosis of GBM patients is generally poor, and there are many types (Verhaak et al., 2010), so the study of its clinical treatment is particularly important.

EGFR VIII is a unique mutant subtype of EGFR in gliomas, and the CRISPR-Cas13a system can induce the death of glioma cells overexpressing EGFR VIII. Single-cell RNA sequencing analysis performed on U87-Cas13a- EGFR VIII cells confirmed that the CRISPR-Cas13a system is characterized by nonspecific RNA cleavage (Wang et al., 2019). It was further shown that the collateral effect of the CRISPR-Cas13a system can be used to kill GBM cells overexpressing EGFR VIII. In addition, CRISPR-Cas13a inhibited glioma formation in intracranial tumors in mice. It has been shown that Cas13a-expressing U87-F3-T3 cells inhibit the growth and proliferation of glioma cells in a mouse tumor model. This revealed that CRISPR-Cas13a inhibition was also observed in tumor xenograft models.

The CRISPR-Cas13a system exerts its powerful tumor elimination potential by exploiting its side effects in glioma cells.

#### 3.6.2 Pancreatic cancer

Pancreatic cancer is a common malignant tumor of the digestive system. Due to its insidious and atypical clinical symptoms, it is a malignant tumor of the digestive tract that is difficult to diagnose and treat. Due to its characteristics, such as low early diagnosis rate, and high surgical mortality, low cure rate and rapid onset, pancreatic cancer is known as the “king of cancer” in the field of cancer. Is the most deadly malignancy of human cancers, so there is an urgent need to develop effective new treatment strategies for this disease (Siegel et al., 2017).

KRAS mutant is a known oncogene driving pancreatic cancer, and inhibition of KRAS mutant expression at the mRNA level is an effective anti-tumor strategy. In experiments, it was found that bacterial Cas13a protein and crRNA significantly knock down the expression of mutant KRAS mRNA (Abudayyeh et al., 2016), confirming that the CRISPR-Cas13a system could induce a knockdown efficiency of up to 94%. The Cas13a-crRNA complex effectively blocks the KRAS-G12D mutation signaling pathway in multiple KRAS-driven pancreatic cancer models, leading to apoptosis and tumor growth inhibition *in vitro* and *in vivo*, demonstrating that the CRISPR-Cas13a system can be used as a targeted therapy for pancreatic cancer mutant KRAS (Zhao et al., 2018).

More importantly, CRISPR-Cas13a-mediated mRNA downregulation can induce apoptosis of mouse tumor cells and cause significant tumor atrophy *in vitro*, and CRISPR-Cas13a knockdown of KRAS-G12D can block the proliferation of pancreatic cancer cells. To develop optimal strategies for the CRISPR-Cas13a system to influence the efficient and specific knockdown of carcinogenic mRNA and establish the CRISPR-Cas13a system as a flexible targeted therapeutic tool.

#### 3.6.3 Cervical cancer

Cervical cancer is a common malignant tumor in women worldwide, and human papillomavirus (HPV) is an important cause of cancer. Cervical cancer is usually associated with multiple types of human papillomavirus (HPV), However, the high-risk human papillomavirus (HR-HPVs) HPV16 and HPV18 are the main causes of cervical cancer (Matsukura and Sugase, 2008). The E6 and E7 oncoproteins, which play a key role in the development of cervical cancer and are encoded by the HPV genome reduce the expression levels of tumor suppressor proteins P53 and RB, which eventually leads to enhanced cell proliferation and reduced apoptosis and promotes the development of cervical cancer (Goodwin and DiMaio, 2000). Therefore, antiviral agents that inhibit the expression of E6/E7 oncoproteins are expected to be a potential treatment for human cervical cancer.

Recent studies have shown that by effectively and specifically knocking down HPV16/18 E6/E7 mRNA using the CRISPR-Cas13a system. In subcutaneous xenograft tumor growth assay, HPV16 knockdown by CRISPR-Cas13a system significantly reduced tumor weight and volume. Studies have shown that targeting HPV E6/E7 mRNA by CRISPR-Cas13a system may be a candidate therapeutic strategy for HPV-related cervical cancer (Chen et al., 2020).

#### 3.6.4 Bladder cancer

Bladder cancer is one of the most common urological malignancies worldwide. Bladder cancer is a heterogeneous disease with a high prevalence and recurrence rate (Kluth et al., 2015).

A novel CRISPR-Cas13a-based light-induced synthesis system can be used to knock down and bind RNA in cancer cells. Moreover, this system can induce the expression of Cas13a, which can be effectively induced under blue light by using a photo sensor in synthetic biology technology (Qi et al., 2019). Long non-coding RNA (LncRNA) can regulate a variety of protein pathways and is believed to play an important and complex role in the occurrence, growth, and progression of tumors (Han et al., 2013). Therefore, the long noncoding RNA MALATI was used as a functional target and tested in bladder cancer 5637 and T24 cells. The results showed that the expression of MALAT1 long non-coding RNA (LncRNA) was decreased in bladder cancer cells (5637 and T24 cells), and the phenotype of bladder cancer was changed under blue light.

The novelty of this study lies in the reversibility and low toxicity of gene editing by using synthetic biology technology to reduce the interference of endogenous genes or proteins.

## 4 Summary and prospect

This review introduces the history, classification, and principle of CRISPR-Cas system.CRISPR-Cas13a belongs to the Class 2 type VI CRISPR-Cas system. Many studies have shown that the Cas13a protein can not only cleave the target RNA but also non-specifically cleave any RNA around it. This property has also been used to develop RNA assays. The application of the CRISPR-Cas13a system in pathogen detection, anti-infection, phage gene editing, agriculture, and gene therapy, among others, and the application of CRISPR-Cas13a in human diseases is also emphasized in the field of RNA editing, such as regulating the expression of harmful genes in the clinical treatment of certain genetic diseases to improve patients*’* conditions.

CRISPR-Cas13a targets RNA, to degrade RNA nonspecifically. When CRISPR-Cas13a binds to the target RNA, it produces nonspecific cleavage RNA, which slows cell growth (Abudayyeh et al., 2017). CRISPR-Cas13a offers a unique way to disrupt the genetic system without interfering with subsequent generations, providing benefits that complement classical genetic methods. Cas13a targets transcripts rather than DNA (Cong et al., 2013). Changing the transcript does not change the DNA itself, making the change reversible and more controllable in time and space than CRISPR-Cas9. The nucleic acid detection technology developed by the CRISPR-Cas13a system has high sensitivity and high specificity. In cancer, the CRISPR-Cas13a system can be used for early cancer detection and rapid detection of tumor-related markers and can be used for targeted therapy of cancer, indicating that this system has considerable application potential.

Despite large advances that have been made the challenges still need to be faced in the aspects of the toxic effect and the off-target effects of the CRISPR-Cas13a system. The toxic effect refers to the non-specific enzyme digestion in bacterial cells that occurs when the HEPN domain of Cas13a catalyzes the digestion of the target ssRNA under the guidance of crRNA, leading to the degradation of other ssRNAs in the cell (Abudayyeh et al., 2016). Cas13-based RNA editing tools target dynamically transcribed RNA, do not cause permanent changes to the genome, and can be controlled through synthetic biology and dose adjustment, which is reversible and relatively safer (Qi et al., 2019). In prokaryotic systems, non-specific degradation of Cas13a occurs. However, in eukaryotic systems, non-specific degradation of CRISPR-Cas13a is highly specific (Cox et al., 2017). Collateral cleavage signals are only found when targeting highly expressed transcripts, i.e. the expression of the target protein must be above a specified threshold to induce non-specific degradation (Bot et al., 2022).

The Off-target effect refers to the mispairing of the designed crRNA with the non-target RNA sequence, thereby introducing unexpected genetic mutations. It has been shown that Cas13a’s target RNA binding affinity and HEPN-nuclease activity are differentially affected by the number and location of crRNA and target RNA mismatches. It also reveals the scope of influence of Cas13a’s RNA binding and cleavage behavior and the possible impact of Cas13a off-target recognition on Cas13a’s ssRNAs and allow rational design of crRNAs with optimal specificity and activity (Tambe et al., 2018). The impact of off-target effects on CRISPR-Cas13a technology was further reduced. Studies have also shown that Cas13a can tolerate single nucleotide mismatch between crRNA and target sequence, but double mismatch can significantly reduce the cleavage efficiency of Cas13a enzyme (Hryhorowicz et al., 2023).

CRISPR-Cas13a is an RNA-mediated gene editing technology targeting RNA. Before the advent of CRISPR-Cas13a, RNA interference (RNAi) technology (Zhou et al., 2013) and CRISPR-Cas9 technology (Sun et al., 2012) were the two main ways to reduce gene expression. RNA interference (RNAi) is an effective way to inhibit gene expression in mammalian cells, mainly acting on the cytoplasm where the RNA-induced silencing complex is located. However, in some cases, RNAi technology is less effective because it does not interact with mRNA still in the nucleus. RNAi technology also further requires fine specificity in the design of small interfering RNA (siRNA), suggesting the need for close to complete identity to the homologous mRNA sequence. These two points limit the application of RNAi technology (Jackson et al., 2003). CRISPR-Cas9 technology has a non-negligible genetic DNA off-target effect. In addition, recent studies have shown that Cas9 toxicity can damage some key genes in cells. These disadvantages greatly limit the clinical application of CRISPR/Cas9 technology (Guo et al., 2023).

Additionally, the application of Cas13a-based genome editing still requires practical identification. Lastly, a major challenge is efficient and tissue-specific delivery, in part because the size of Cas13-based RNA editors developed to date exceeds the packaging capacity of adeno-associated viruses (AAV). The use of smaller Cas13a homologs and the small size of the Cas13 protein offer new opportunities for programmable RNA modulation, particularly *in vivo*. Cas13 homologs, compatible with the delivery of AAV, further promote the development of programmable RNA editing techniques (Kannan et al., 2022). Recent research has developed DIRECTED platforms for point-to-point delivery of specific cargoes and target cells (Strebinger et al., 2023). In addition, the long-term effects of Cas13a expression in heterologous eukaryotes still need to be investigated.

Finally, the potential for editing the human genome and the possibility of using Cas13a-based gene drives for ecosystem engineering raised ethical issues that must be considered. Even so, the current pace of scientific advances related to Cas13a and the results that can be obtained using the simplest gene drives suggest that editing the genomes of wild populations by CRISPR-Cas13a will be realized by molecular biologists shortly. At present, much progress has been made in the application of CRISPR-Cas13a technology, but further exploration is still needed before it can develop into a mature technical system.

## Author contributions

LZ: Supervision, Writing – review & editing, Funding acquisition, Project administration. SL: Conceptualization, Data curation, Visualization, Writing – original draft. RL: Conceptualization, Investigation, Visualization, Writing – review & editing. XQ: Conceptualization, Investigation, Visualization, Writing – review & editing. TF: Investigation, Visualization, Writing – review & editing. BW: Writing – review & editing. BZ: Writing – review & editing. YZ: Conceptualization, Investigation, Validation, Visualization, Writing – original draft, Writing – review & editing.
