# Erratum: Advances in application of CRISPR-Cas13a system

**DOI:** 10.3389/fcimb.2024.1393044

**Published:** 2024-03-08

**Authors:** Frontiers Production Office

**Affiliations:** Frontiers Media SA, Lausanne, Switzerland

**Keywords:** CRISPR-Cas system, CRISPR-Cas13a, genome editing, molecular diagnosis, gene therapy

Due to a production error, the positions of Li Zhang and Yue Zhang were misplaced in the author list, which should be as follows:

Yue Zhang^1†^, Shengjun Li^2†^, Rongrong Li^3^, Xu Qiu^1^, Tianyu Fan^4^, Bin Wang^1^, Bei Zhang^1^ and Li Zhang^1*^.

The publisher apologizes for this mistake.

The original version of this article has been updated.

